# Factors affecting gestation periods in elasmobranch fishes

**DOI:** 10.1242/bio.059270

**Published:** 2022-06-10

**Authors:** Soma Tokunaga, Yuuki Y. Watanabe, Mai Kawano, Yuuki Kawabata

**Affiliations:** 1 Faculty of Fisheries, Nagasaki University, Bunkyo, Nagasaki 852-8521, Japan; 2 Department of Polar Science, The Graduate University for Advanced Studies, SOKENDAI, Tachikawa, Tokyo 190-8518, Japan; 3 National Institute of Polar Research, Tachikawa, Tokyo 190-8518, Japan; 4 Faculty of Agriculture, Kyushu University, Nishi-ku, Fukuoka 819-0395, Japan; 5 Atmosphere and Ocean Research Institute, The University of Tokyo, Kashiwa, Chiba 277-8564, Japan; 6 Graduate School of Fisheries and Environmental Sciences, Nagasaki University, Bunkyo, Nagasaki 852-8521, Japan

**Keywords:** Endothermy, Metabolism, Phylogenetic analysis, Scaling, Viviparity

## Abstract

Gestation periods vary greatly across elasmobranch species. Differences in body size and body temperature (i.e. major determinants of metabolic rates) might explain such variation. Although temperature effects have been demonstrated for captive animals, body size effects remain undocumented. Moreover, whether metabolic rates of mothers or those of embryos affect gestation periods remains unclear. Because biological times generally scale with mass^1−*β*^, where *β* is metabolic scaling exponent (0.8–0.9 in fishes), we hypothesized that elasmobranch gestation periods would scale with mass^0.1–0.2^. We also hypothesized that regionally endothermic species with elevated metabolic rates should have shorter gestation periods than similar-sized ectothermic species if the metabolic rates of mothers are responsible. We compiled data on gestation periods for 36 elasmobranch species to show that gestation periods scale with *M*^0.11^ and *m*^0.17^, where *M* and *m* are adult female mass and birth mass, respectively. Litter size and body temperature also affected gestation periods. Our findings suggest that the body-mass dependence of metabolic rate explains some variations in elasmobranch gestation periods. Unexpectedly, regionally endothermic sharks did not have shorter gestation periods than their ectothermic counterparts, suggesting that the metabolic rates of embryos, which are likely ectothermic in all elasmobranch species, may be responsible.

This article has an associated First Person interview with the first author of the paper.

## Abstract (precis)

**Summary:** Interspecific variations in elasmobranch gestation periods can largely be explained by body-mass dependence of metabolic rate.

## INTRODUCTION

Gestation periods, or the duration of pregnancies, are a crucial feature of animals' reproductive strategies and affect population dynamics. Viviparous (i.e. producing living young) species are found in all major vertebrate groups except for birds (Rothchild, 2003). Species-specific gestation periods and factors affecting them have long been studied primarily in mammals (Martin and MacLarnon, 1985; Martin, 1996; Sacher and Staffeldt, 1974; Weisbecker and Goswami, 2010). Elasmobranch is another taxonomic group that has many viviparous species. Their gestation periods vary among species from a few months to years (Furumitsu et al., 2019; Hanchet, 1988). However, factors causing this variation are poorly understood. Each species is thought to have evolved specific gestation periods, so that fitness could be maximized under given ecological, physiological, and environmental constraints. Therefore, clarifying factors affecting elasmobranch gestation periods helps better understand the reproductive strategies, population dynamics, and evolutionary history of this group of animals.

Warm waters have been shown to facilitate embryonic growth and shorten gestation periods in elasmobranchs. Dogfish *Scyliorhinus canicula* exhibit twice higher embryonic growth rates when kept in waters that are 4°C warmer (Harris, 1952). Eagle rays *Aetobatus narinari* have a 180–188 day gestation period at 28.1–30.1°C water temperature, and this period increases to 328–399 days in cooler waters (23.5–24.5°C) (Swider et al., 2017). Moreover, many elasmobranch females seasonally migrate to warmer waters, presumably to facilitate embryo growth and shorten gestation periods (Bernal et al., 2012; Hight and Lowe, 2007; Jirik and Lowe, 2012). These temperature effects suggest that metabolic rate, the rate at which organisms produce energy, affects embryo growth rates and thus gestation periods. In addition to body temperature, metabolic rates are a function of body mass (Peters, 1983); however, the effects of body mass on elasmobranch gestation periods have never been investigated. Metabolic rate *Y* is proportional to mass*^β^*, where *β* is a metabolic scaling exponent (Peters, 1983). In general, biological times (e.g. life span and population doubling time) are proportional to mass^1−*β*^ (Lindstedt and Calder, 1981; Peters, 1983). Because *β* is approximately 0.8–0.9 in fishes (Clarke and Johnston, 1999; White et al., 2006), including sharks (0.84; Ste-Marie et al., 2020), we would predict that gestation periods of elasmobranchs scale with mass^0.1–0.2^.

Given that metabolic rates affect gestation periods in elasmobranchs, a question arises: are metabolic rates of mothers or those of their embryos more important? If gestation period *t* is largely determined by the mother's biomass production rate or metabolic rate, *t* would be expressed as *t*∝*M*^0.1–0.2^, where *M* is mother's body mass. In a different scenario, consider that embryo growth rates are affected by the embryos' own metabolic rates, given that they receive sufficient nutrition and oxygen from their mother. In this case, the ontogenetic growth model would apply, where gestation period *t* is expressed by birth mass *m* as *t*∝*m*^1−*β*^ (Moses et al., 2008; West et al., 2001), that is, *t*∝*m*^0.1–0.2^ in elasmobranchs. Therefore, we would predict that elasmobranch gestation periods scale with *M*^0.1–0.2^ or *m*^0.1–0.2^, depending on whether the metabolic rates of mothers or embryos play a more important role.

Another important factor to consider is thermal physiology. Most elasmobranch species are ectotherms, with their body temperature being close to the ambient water temperature. Yet, regionally endothermic species (e.g. white sharks) maintain elevated body temperature by using the heat produced by continuous swimming activities (Bernal et al., 2012; Lowe and Goldman, 2001). They have higher metabolic rates than similar-sized ectothermic species, even with the difference in body temperature taken into account (Carlson et al., 2004). On the other hand, their embryos are unlikely to have elevated metabolic rates, given that embryos cannot produce heat by continuous swimming activities. Therefore, if metabolic rates of mothers, rather than embryos, affect gestation periods, regionally endothermic species should have shorter gestation periods than ectothermic species for a given mass and body temperature. Furthermore, litter size (i.e. the number of offspring produced at one parturition) varies among species from a few to hundreds in elasmobranchs (Cortés, 2000; Joung et al., 1996; Nielsen et al., 2020). This variation may affect maternal investments per embryo and thus gestation periods.

In this study, we compiled data on elasmobranch gestation period from the literature and examined how the variations in gestation periods can be explained. Specifically, we examined (1) whether gestation period scales with *M*^0.1–0.2^ or *m*^0.1–0.2^ and (2) whether the regionally endothermic sharks have shorter gestation periods than ectothermic species for a given mass and body temperature.

## RESULTS

**Fig. 1.**
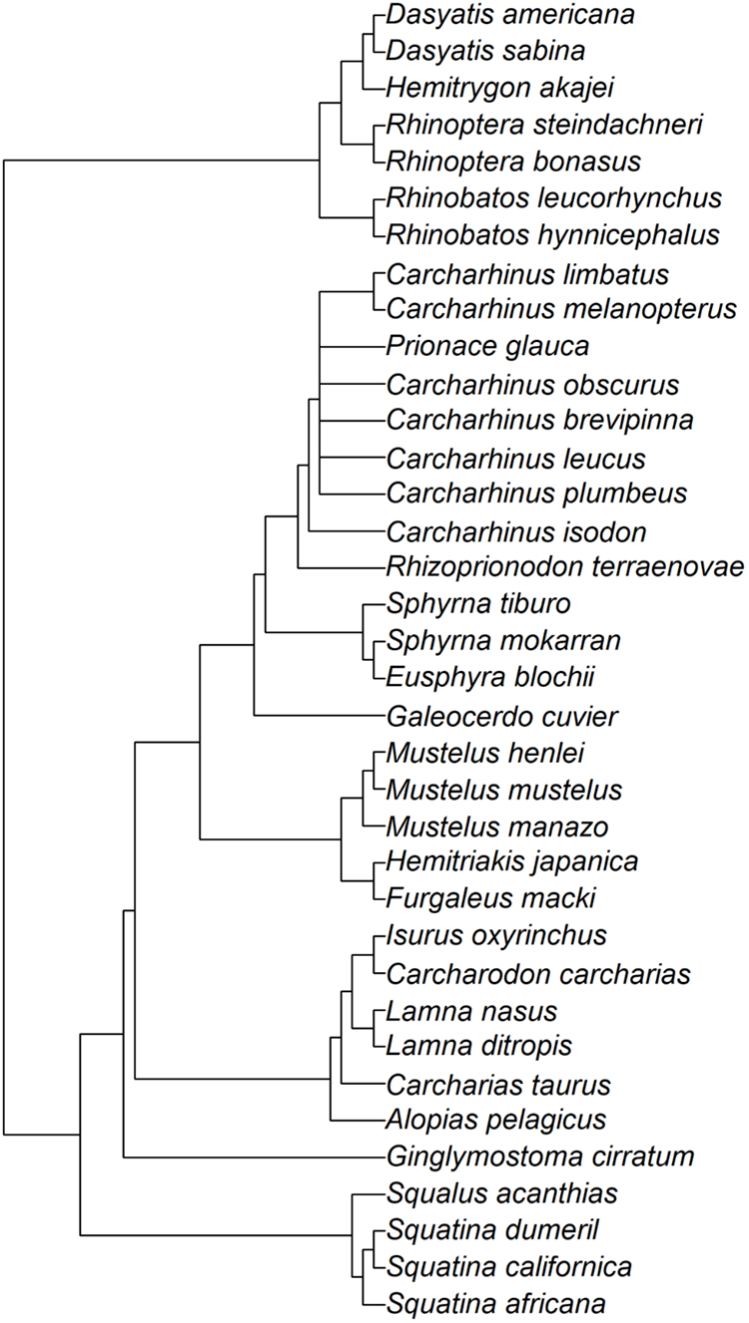
Phylogenetic tree used for PGLS analysis.

**Fig. 2.**
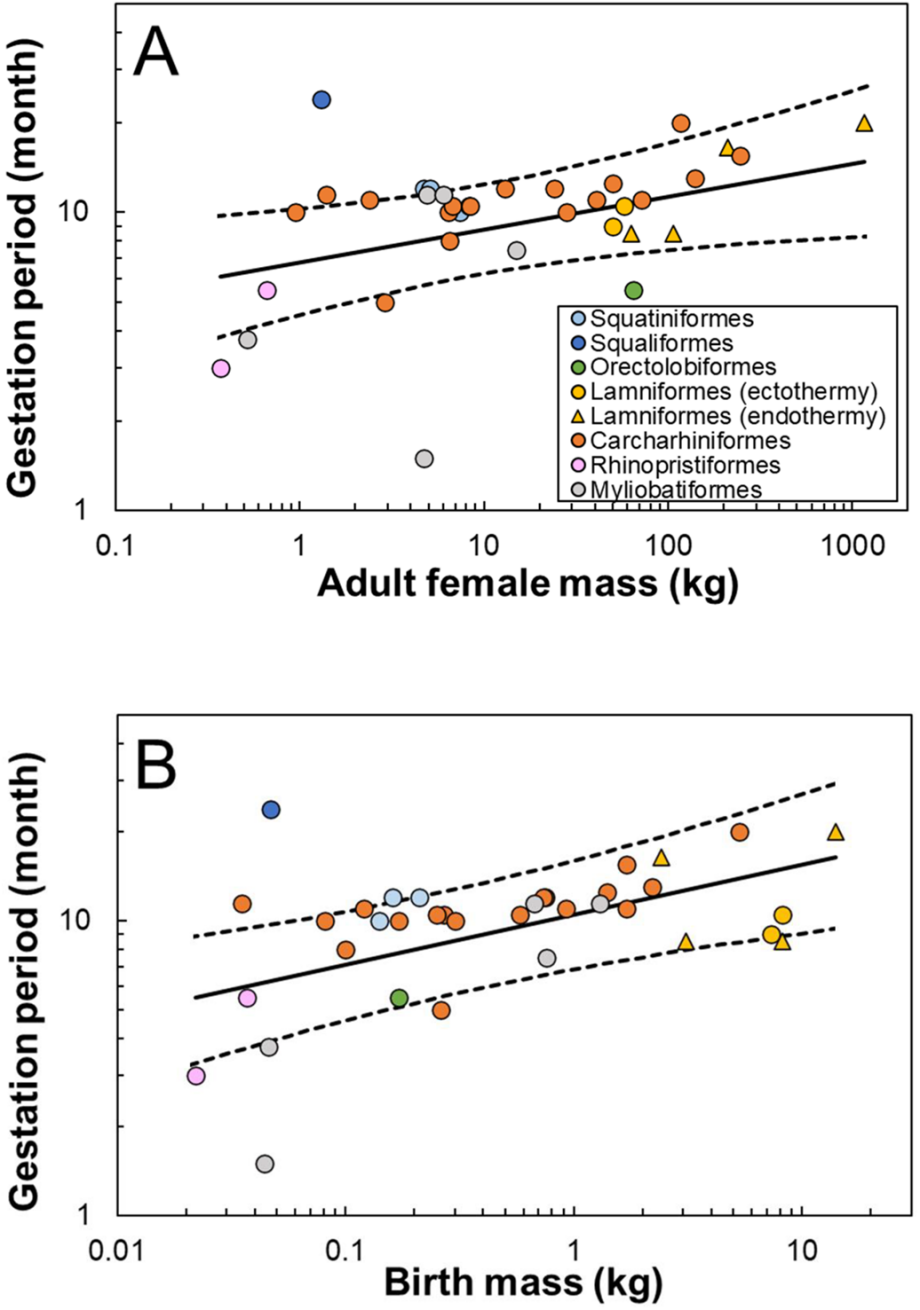
**Gestation period *t* plotted against (A) adult female mass *M* and (B) birth mass *m*.** Solid lines represent the PGLS regression lines [(A) *t*=6.8×*M*^0.11^; (B) *t*=10.5×*m*^0.17^]. Dashed lines represent the 95% confidence interval of the regression lines. Plots were colored by order to visualize phylogenetic relationships.

We compiled data on gestation periods for 36 elasmobranch species, spanning from 0.37–1159 kg in adult female mass, including four regionally endothermic species (Table1). Fig. 1 shows a phylogenetic tree used for our phylogenetically-informed analyses (see Materials and Methods). Gestation periods *t* ranged from 1.5 to 24 months (Table 1) and increased with adult female mass *M* as *t*=6.8×*M*^0.11^ (Fig. 2A) and birth mass *m* as *t*=10.5×*m*^0.17^ (Fig. 2B). The scaling exponents fell within the predicted range (0.1–0.2) both for *M* (0.11; 95% confidence interval 0.01–0.21) and *m* (0.17; 95% confidence interval 0.07–0.28).

**Table 1.**
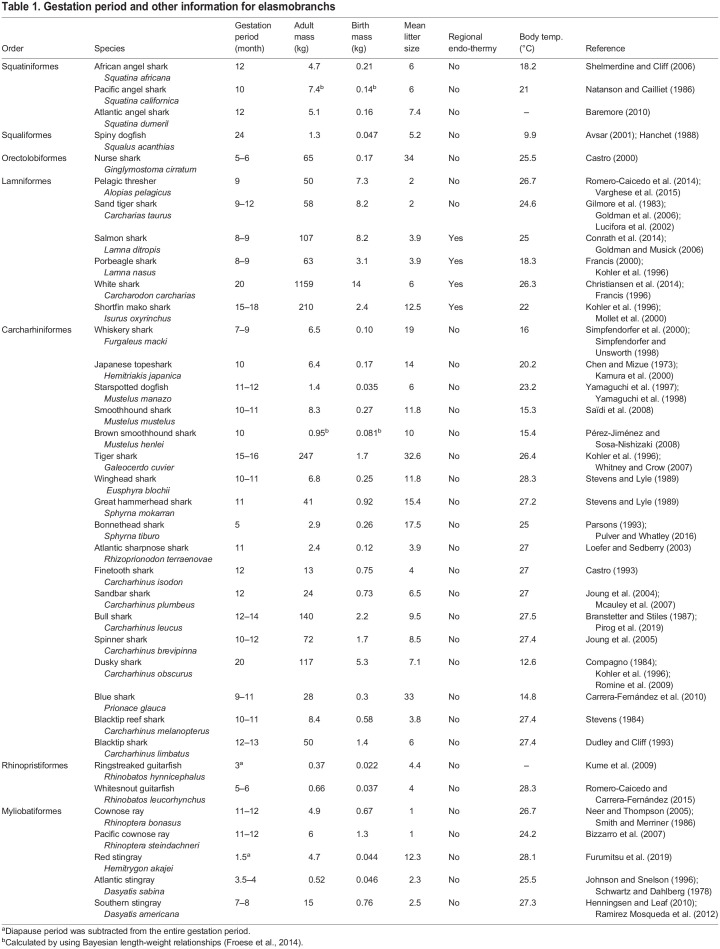
Gestation period and other information for elasmobranchs

**Fig. 3.**
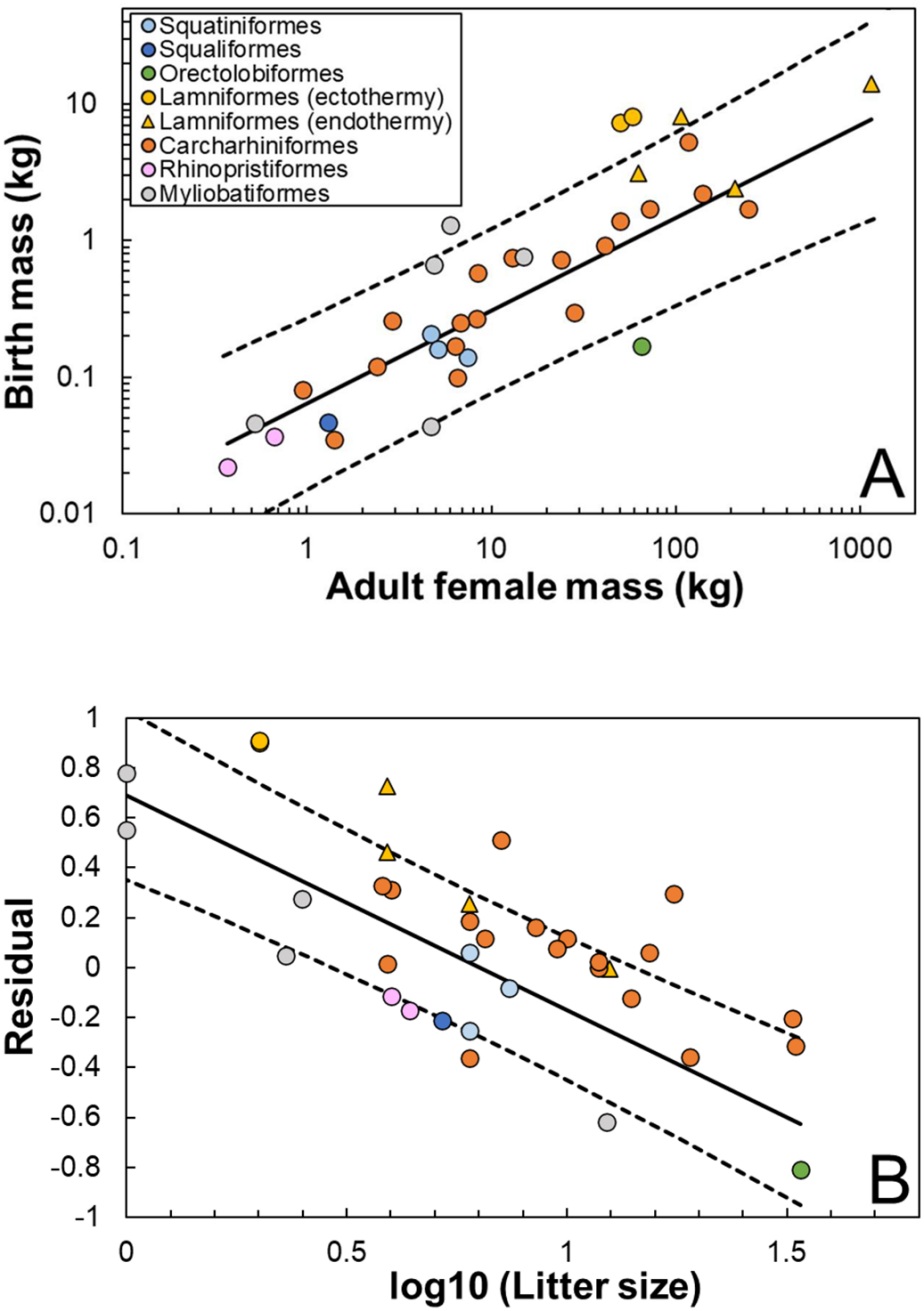
**Relationship between relative birth mass and mean litter size *L*.** (A) Relationship between adult female mass *M* and birth mass *m*. Solid line represents PGLS regression line: *m*=0.064×*M*^0.68^ (R^2^=0.60). (B) Relationship between residuals calculated from the regression line in A [i.e. log_10_ (observed *m*)–log_10_ (predicted *m*)] and log_10_ (*L*). Solid line represents PGLS regression line: Residual=−0.86×log_10_ (*L*)+0.69 (*R*^2^=0.60). Dashed lines represent the 95% confidence interval of the regression lines.

When *M*, *m*, and whether the species has regional endothermy were inputted as predictor variables, the best model included *m* rather than *M*. The effect of regional endothermy was not included in the best model. When litter size *L* was added as a predictor variable, the best model included *M* and *L* as *t*=10.6×*M*^0.16^×*L*^−0.32^ (Table 2). The partial regression coefficient for *L* was negative (−0.32), meaning that gestation periods decrease as litter size increases. Birth mass increased with adult female mass as *m*=0.064×*M*^0.68^ (*R*^2^=0.60) (Fig. 3A). Residuals of this relationship [i.e. log_10_ (observed *m*)–log_10_ (predicted *m*)] were negatively associated with log_10_ (*L*) as: Residual=−0.86×log_10_ (*L*)+0.69 (R^2^=0.60) (Fig. 3B).

**Table 2.**
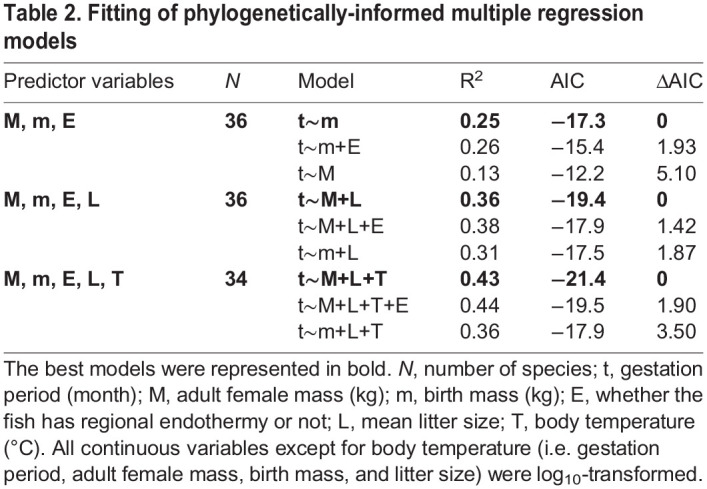
Fitting of phylogenetically-informed multiple regression models

We compiled data on body temperature *T* (i.e. preferred water temperature for ectothermic species and reported body temperature for regionally endothermic species) for 34 out of 36 species (Table 1). Gestation periods were best explained by *M*, *L*, and *T* as *t*=26.9×*M*^0.16^×*L*^−0.33^×10^−0.017*T*^ (Table 2). The partial regression coefficient for *T* was negative (−0.017; 95% confidence interval −0.004 – −0.029), indicating that gestation period decreases as body temperature increases.

## DISCUSSION

Consistent with our hypothesis, elasmobranch gestation periods scaled with mass^0.1–0.2^, suggesting that body mass (adult female mass and birth mass) affects gestation periods through its effects on metabolic rates. Large variations around the regression lines are partly explained by the variations in litter size and body temperature, as shown in our analyses. Furthermore, viviparous elasmobranchs exhibit various reproductive modes (e.g. ovoviviparous, placental viviparous), which may also influence gestation periods. Depending on the mode, embryos receive nutrition from their mother in different forms, such as yolk-sacs, placental transfer, uterine secretions, ova, or siblings (Hamlett et al., 2005; Parsons et al., 2008). However, reproductive modes are still uncertain for many species, and some rely on a combination of multiple nutrient sources (Cotton et al., 2015; Sato et al., 2016). These complexities precluded us from modeling the effect of different reproductive modes in this study. Nevertheless, we found a robust result that elasmobranch gestation periods scaled with mass^0.1–0.2^.

Gestation periods decreased as body temperature increased among different elasmobranch species. This result is consistent with previous studies that examined the effect of water temperature on gestation period with captive individuals (Harris, 1952; Swider et al., 2017). The 95% confidence interval for the regression coefficient (i.e. −0.004 – −0.029) indicates that a 10°C decrease in body temperature is associated with 1.1–1.9-times longer gestation periods within the temperature range covered by our dataset (9.9–28.3°C; Table 1). This value is lower than the temperature dependence of metabolic rate in elasmobranch with an interspecific Q_10_ of 2.23 (95% confidence interval 1.74–2.85; Ste-Marie et al., 2020), possibly due to errors in our body temperature estimates (discussed below).

Unexpectedly, regionally endothermic sharks did not have shorter gestation periods than their ectothermic counterparts. Regionally endothermic sharks have higher metabolic rates than ectothermic sharks for a given mass and body temperature (Carlson et al., 2004), allowing them to cruise at faster speeds and migrate longer distances (Watanabe et al., 2015). By contrast, their embryos are likely ectothermic without elevated metabolic rates because embryos cannot produce metabolic heat by continuous swimming activities. Thus, our results suggest that metabolic rates of embryos may have stronger effects on gestation periods than those of mothers. Yet, we need to be cautious, because direct measurements of embryo body temperature or metabolic rates are currently lacking. The possibility that even embryos have elevated metabolic rates (via maternal investment or innately) cannot be ruled out.

The best model included adult female mass, rather than birth mass when litter size was added as a predictor variable (Table 2). The negative regression coefficient indicates that gestation periods decrease as litter size increases. This finding can be explained by our observation that larger litter size is associated with smaller birth mass relative to adult mass (Fig. 3). An ontogenetic growth model (Moses et al., 2008; West et al., 2001) predicts that smaller relative birth mass leads to shorter gestation periods unless *M*≫*m* (for details, see Moses et al., 2008). Our results provide support for this prediction. The ontogenetic growth model assumes that embryo growth rates are not limited by maternal investment. Therefore, our analyses of litter size further suggest that the metabolic rates of embryos, rather than those of their mothers, may affect elasmobranch gestation periods.

We acknowledge several limitations of this study. First, we were unable to directly compare gestation periods with metabolic rate due to the sparseness of metabolic rate data in elasmobranch. Second, although we considered that metabolic rate affects growth rate, causality may be the reverse, or body mass and body temperature may influence growth rate independently of metabolic rate (Glazier, 2015). Third, the metabolic scaling exponent *β* may vary between 0.67 and 1 depending on ecological lifestyle and activity level of organisms (Glazier, 2010). Fourth, our estimate of body temperature for each species (see Materials and Methods) may be inaccurate. For example, because sexual segregation is common in elasmobranch (Wearmouth and Sims, 2008), preferred water temperature for pregnant females may be different from that reported for the species in general. Therefore, further research is needed to better understand the association between metabolic rates and elasmobranch gestation periods. In mammals, allometric relationships between gestation period and body mass are consistent with the metabolism framework (i.e. *t* scales with mass^1−*β*^) in some studies (Blueweiss et al., 1978; Sacher and Staffeldt, 1974), but not others (Clauss et al., 2014; Western and Ssemakula, 1982).

In conclusion, our comparative analyses show that elasmobranch gestation periods scaled with mass^0.1–0.2^, suggesting that the body-mass dependence of metabolic rate can explain some variations in gestation periods. Litter size and body temperature also affect gestation periods. Unexpectedly, regionally endothermic sharks did not have shorter gestation periods than their ectothermic counterparts. Metabolic rates of embryos, which are likely ectothermic in all elasmobranch species, may have stronger effects on gestation periods than those of their mothers. The empirical relationships shown in this study provide a new perspective for understanding the life history of elasmobranchs.

## MATERIALS AND METHODS

### Data compilation

We compiled data on gestation periods (in months) for as many elasmobranch species as possible from the literature. Gestation periods have been estimated for captive and wild individuals. However, captive individuals likely have different nutritional conditions and body temperature from wild individuals, presumably leading to different embryo growth rates and gestation periods. Because we aimed to understand elasmobranch natural life histories, we only compiled data on wild individuals. The following three methods have been used to estimate elasmobranch gestation periods in the wild: (1) plotting the sampled embryo size against the time of year for a species and estimating the duration required for embryo to grow to birth size; (2) estimating mating season and pupping season and calculating the difference between the two; (3) extrapolating length-age relationships to the point where body length is zero. We considered the first method most accurate and accepted the literature that used this method. In the second method, embryo growth rates are uncertain, and whether gestation periods are less than a year or more than a year remains a question. In the third method, embryo growth rates are uncertain. When multiple studies were found for a species, we accepted the study with the largest sample size. Regional variations within species (Lombardi-Carlson et al., 2003; Yamaguchi et al., 2000) were not considered in this study. In our dataset, ringstreaked guitarfish *Rhinobatos hynnicephalus* and red stingray *Hemitrygon akajei* appear to exhibit embryonic diapause. During this diapause period, uterine eggs without macroscopic embryos are observed, and embryonic growth begins after this period (Furumitsu et al., 2019; Kume et al., 2009). Hence, for these species, we estimated gestation periods as the length between the beginning and completion (i.e. parturition) of embryonic growth by subtracting the diapause period from the entire gestation period.

For each species in our dataset, female body mass at maturity (kg) was estimated based on information on body length at maturity and length-weight relationships found either in the literature or FishBase (http://www.fishbase.org. Version 02/2021). When body length at maturity was reported as a range, we used the middle value. Birth mass (kg) was also compiled based on the previously documented birth size, the largest embryo size, or the smallest neonate size combined with length-weight relationships. Regionally endothermic species was identified following Dickson and Graham (2004). The mean litter size was compiled from the same literature as that which reported gestation periods. When litter size was reported as a range, we used the middle value. As an approximation of body temperature, the mean preferred water temperature was compiled for each ectothermic species using FishBase. For regionally endothermic species, the mean body temperature measured for free-swimming fish using bio-telemetry or for freshly caught specimen was compiled (Carey et al., 1981; Goldman, 1997; Goldman et al., 2004; Sepulveda et al., 2004).

### Statistical analyses

We examined the allometric relationships between gestation period and body mass (i.e. adult female mass and birth mass) by using the Phylogenetic Generalized Least Squares (PGLS) method, which controls for phylogenetic non-independence of each species' traits (Lavin et al., 2008). We created a phylogenetic tree using the software Mesquite with the phylogenetic relationships based on Chondrichthyan Tree of Life website (https://sharksrays.org/; accessed 20 December 2020) and an arbitrary branch length (Grafen, 1989). Then, we computed the PGLS regressions with their 95% confidence intervals using the software R with ‘caper’, ‘evomap’, and ‘geiger’ package (Smaers and Rohlf, 2016). We used Pagel's λ to maximize the model's likelihood (Freckleton et al., 2002).

Multiple regression analyses were performed with gestation periods as the response variable. Predictors included continuous variables (i.e. adult female mass, birth mass, litter size, and body temperature) and a categorical variable (i.e. regional endothermy or ectothermy). Adult female mass was strongly correlated to birth mass, posing a risk of collinearity; therefore, we only used these variables in different models. All continuous variables except for body temperature (i.e. gestation period, adult mass, birth mass, and litter size) were log_10_-transformed to improve the linearity of relationships among the variables. Plots were checked visually to ensure that model assumptions (i.e. homogeneity of variance, normality of error) were reasonably met. The best models were selected based on Akaike Information Criteria (AIC). In our hypothesis-driven analyses, interactions among predictor variables were not considered.
